# Application of Modified Seed Oils of Selected Fruits in the Synthesis of Polyurethane Thermal Insulating Materials

**DOI:** 10.3390/ma17010158

**Published:** 2023-12-28

**Authors:** Elżbieta Malewska, Maria Kurańska, Maria Tenczyńska, Aleksander Prociak

**Affiliations:** Department of Chemistry and Technology of Polymers, Cracow University of Technology, Warszawska 24, 31-155 Cracow, Poland; maria.tenczynska@student.pk.edu.pl (M.T.); aleksander.prociak@pk.edu.pl (A.P.)

**Keywords:** biopolyol, seed oil, biofoam, polyurethane, thermal insulating materials

## Abstract

The use of alternative raw material sources in polyurethane chemistry is necessary given the limited supply of fossil fuels, their rising prices and the concern for sustainability. The production of biopolyols from edible vegetable oils such as rapeseed oil, soybean oil or sunflower oil is often proposed. In order to avoid conflict with the global food economy, non-edible or waste oils are hoped to find application in chemical synthesis. The possibility of using oils from selected fruit seeds to obtain biopolyols is analyzed in this manuscript. Five biopolyols were obtained from watermelon, cherry, black currant, grape and pomegranate fruit seeds using the transesterification reaction of the oils with triethanolamine. Thermal insulating polyurethane foams were then obtained by replacing 75% of petrochemical polyol with the biopolyols in polyurethane systems. Based on an analysis of the foaming process, it was found that the incorporation of triethanolamine molecules into the biopolyols causes a catalytic effect. The use of such biopolyols allows eliminating the catalyst from a polyurethane foam formulation. The polyurethane biofoams obtained with the pomegranate-seed-based biopolyol were characterized by the highest content of closed cells (45 vol.%). The lowest content was found for the foams containing the currant-seed-based biopolyol (9%). The foams were characterized by thermal conductivity coefficients between 32 and 35 kW/m·K and densities of approximately 40 kg/m^3^. Good dimensional stability and compressive strength between 100 and 250 kPa make them suitable for use in construction.

## 1. Introduction

The plastics industry is one of the main industrial sectors in Europe. In 2021, the global plastic production amounted to 390.7 million tons, with the European production limited to 57.2 million tons [1]. Environmentally friendly technologies play an increasingly important role in the production of plastics. One of the latest initiatives of the European Union related to environmental protection, which also concerns the development of polymers, is to achieve climate neutrality by 2050.

Climate neutrality involves a balance between emissions and the removal of carbon dioxide from the atmosphere. As part of the “Fit for 55” project, the European Union also aims to reduce greenhouse gas emissions by 55% by 2030. To achieve the goals included in the “Fit for 55” package, it is important to use energy from renewable sources on a large scale and implement energy-saving technologies, including polymer technologies consistent with the principles of green chemistry.

Nowadays, it is necessary to reduce carbon dioxide emissions. One method is to use biomass as a source to produce polymeric materials. Vegetable oils, lignin and sugars can be used to produce biomass-based polyols [2]. In order to produce biopolyols from biomass, chemical modification or biological conversion is necessary depending on the type of the starting material chosen and the expected properties [3,4,5].

Biomass includes vegetable oils, which can be effectively used during the synthesis of a new class of polyols and then used to produce thermal insulation materials like PUR foams [6]. Additionally, these oils have a number of advantages, such as easy availability, non-toxicity, renewability and the ability to carry out chemical modifications. Vegetable oils can be chemically modified in several ways, i.e., by epoxidation of the double bond combined with oxirane ring opening by a suitable agent [7], hydroformylation [8], transesterification [9], transamidation [10] or ozonolysis [5]. It is also important to ensure that the use of vegetable oils in chemical synthesis does not disrupt the world’s food economy. Commonly available edible oils, i.e., rapeseed oil [11], sunflower oil [12] and soybean oil [13], are often used in ongoing research to synthesize components for the production of polyurethanes. A new challenge is the search for non-edible vegetable oils [14] or waste oils [15] that could be used as alternative renewable sources.

Another important source of raw materials for polyurethanes (PUR) is recycling. Recycling reduces the amount of waste accumulated in landfills and turns it into valuable raw material used in chemical synthesis. Recyclates obtained from PUR foams [16], as well as other plastics, such as the recently very popular polylactide [17], polyethylene terephthalate [18] and waste tires [19], have found applications in the manufacture of PUR thermal insulation materials. The amount of research undertaken in order to identify promising alternatives to fossil raw materials indicates how important the topic is. The stability of the plastics market is positively influenced by the diversity of raw materials used in their synthesis.

More than one billion tonnes of different fruits are produced annually worldwide. This large production leads to the generation of by-products and waste. In Europe, it is estimated that about 1 million tonnes of waste are produced annually by the fruit and vegetable industry [20]. These large volumes of waste, when properly processed, could provide a low-cost raw material rich in potentially valuable components for other industries. Such waste is also rich in many substances such as vitamins, minerals, lipids, proteins, carbohydrates and polyphenolic compounds [21]. Some fruit seeds contain a lot of oils that can be easily modified chemically. Depending on the type of plant, the seeds can contain up to 50% oil [22]. In the literature, there are reports on efforts made to obtain polyurethane materials from primarily grape seed oil [23,24,25]. Polyols for this purpose were obtained by epoxidation of the double bond and opening oxirane rings.

The present work describes another approach to obtaining thermal insulating polyurethane materials. In our research, a decision was made to test the possibility of using oils from seeds of pomegranate, grape, cherry, black currant and watermelon as potential sources of renewable raw materials. The oils underwent a transesterification process. This type of research on the possibility of applying biomass from fruit waste has not been described in the literature so far.

## 2. Materials and Methods

Watermelon, grape, blackcurrant, pomegranate and cherry seed oils were purchased from OlVita, Poland in the form of cold-pressed oils. In the transesterification reaction, triethanolamine (TEA) (Avantor Performance Materials Poland, Gliwice, Poland) as a transesterification agent and anhydrous zinc acetate (Chempur, Piekary Śląskie, Poland) as a catalyst were used. Biopolyols were synthesized using the transesterification method of watermelon, cherry, pomegranate, grape and blackcurrant seed oils with triethanolamine. The selection of transesterification reaction conditions was based on the previously conducted experiments [26]. The transesterification process was carried out at a temperature of 175 °C for 2 h. Oil, catalyst and TEA were placed in the reactor and mixed mechanically. The molar ratio of oil to TEA was 1:3. The catalyst was added in an amount of 0.3% by mass in relation to the total mass of the oil and TEA.

The oils and biopolyols were evaluated using the following methods. Iodine value (I_val_) analysis was performed using the Hanus method according to the PN-87/C-04281 standard. Hydroxyl value (OH_val_) was found in accordance with the PN-C-89052:1993 standard. The number average molecular weight (M_n_), weight average molecular weight (M_w_) and dispersity (D) of the biopolyols were found using a gel permeation chromatography (GPC) analysis. The analysis was carried out at 35 °C. Tetrahydrofuran was used as an eluent and the flow rate was set to 1 mL/min. The chemical structures of the oil and biopolyols were analyzed using a Nicolet iS5 FTIR spectrometer (Thermo Fisher Scientific, Waltham, MA, USA), equipped with an ATR attachment and a diamond crystal. Spectra were recorded in the infrared range of 4000–500 cm^−1^. Additionally, viscosity at 25 °C using a Lamy Rheology CP-4000 was measured.

In order to obtain polyurethane foams, various raw materials were used in addition to the biopolyols. Rokopol ^®^ RF551 (PCC Rokita SA., Brzeg Dolny, Poland), a sorbitol polyether polyol with a molecular weight of 600 g/mol, functionality of 4.5, and hydroxyl number of 400–440 mgKOH/g, was used as a petrochemical polyol. Polymeric 4,4’-diphenylmethane diisocyanate (produced by Minova Ekochem Sp. z o.o., Siemianowice Śląskie, Poland) with an NCO content of 31 wt% was applied as an isocyanate component. In addition, the surfactant Niax Silicone SR-321 (Momentive Performance Materials, Wilton, Connecticut, USA) and distilled water as chemical foaming agent were used.

A detailed formulation of the manufactured rigid PUR foams is presented in Table 1. The liquid reaction mixture was poured into an open mold to rise freely.

The foaming process was analyzed using a FOAMAT^®^ device (manufactured by Format Messtechnik GmbH, Karlsruhe, Germany). During the foaming process, the temperature change in the foam core and the changes in dielectric polarization were monitored. In addition, the characteristic times of the foaming process, i.e., start, gelation and dry times, were measured.

The foam materials obtained in the molds were seasoned for 24 h and then cut into suitable samples and subjected to tests of physical and mechanical properties. Each type of foam material was obtained three times. The thermal conductivity coefficient at an average temperature of 10 °C (the temperature of the cooling plate was 0 °C and that of the heating plate was 20 °C) was found using a Laser Comp Heat Flow Instrument FOX 200 constructed in accordance with ISO 8301. Samples having dimensions of 20 × 20 × 5 cm were used in this analysis. 

In the next stage, the cellular structure of the materials was analyzed. For this purpose, the percentage content of closed cells was determined in accordance with ISO 4590. Four 25 × 25 × 100 mm samples were cut out of each foam material. The cell structures were analyzed using a HITACHI S-4700 scanning optical microscope equipped with a NORAN Vantage microanalysis system.

The apparent density of the foam materials was determined in accordance with the standard ISO 845. Compressive strength tests were carried out according to EN ISO 844, both perpendicular and parallel to the foam growth direction. Eight cylindrical samples with a diameter of 40 mm and a height of 40 mm were prepared from each material and tested using a Zwick Roell Z005. The compressive strength of the samples was measured at a strain of 10% and a compression speed of 2 mm/min.

The brittleness of the rigid PUR foams was tested according to ASTM C-421-61. Brittleness was determined for 12 cubes with dimensions of 20 × 20 × 20 mm cut out of the analyzed material. Water absorption analysis was carried out according to EN 12088 at room temperature for samples with dimensions of 100 × 100 × 25 mm.

## 3. Results

The oils selected for transesterification reactions were subjected to basic analyses, the results of which are summarized in Table 2. For all analyzed oils, the iodine value was higher than 100 gI_2_/100 g of oil, indicating the presence of double bonds in the chains of fatty acid residues. The oils were characterized by similar average molar masses (Mn) ranging from 822 to 865 g/mol. For most oils, the viscosity was approximately 50 mPa∙s. Only pomegranate oil had a higher viscosity of 200 mPa∙s. Based on the average molar masses of the oils, the amount of TEA necessary to carry out the transesterification reaction was calculated.

To complete the transesterification reaction, 3 moles of TEA are required in relation to 1 mole of oil. The transesterification reaction is an equilibrium reaction that occurs in stages. As a result of the reaction of oil with TEA, diglycerides, monoglycerides, monoesters, diesters, triesters and glycerin may be produced [11,27,28]. The selected reaction conditions in accordance with previously conducted research shifted the reaction balance toward obtaining an increased yield of monoglyceride and monoester.

As a result of the reactions carried out, five different biopolyols were obtained and described. Table 3 shows the basic properties of the biopolyols obtained from various fruit seed oils.

The biopolyols obtained were characterized by similar hydroxyl number values of between 355 and 381 mgKOH/g of polyol. The OH_val_ was taken into account when calculating formulations for the preparation of polyurethane foams. Among the biopolyols obtained, the polyol derived from pomegranate oil had the highest Mn and the highest viscosity. This may be related to the highest Mn and viscosity of the starting oil. The other biopolyols were characterized by Mn of about 280 g/mol and a viscosity of about 160 mPa∙s.

**Figure 1:**
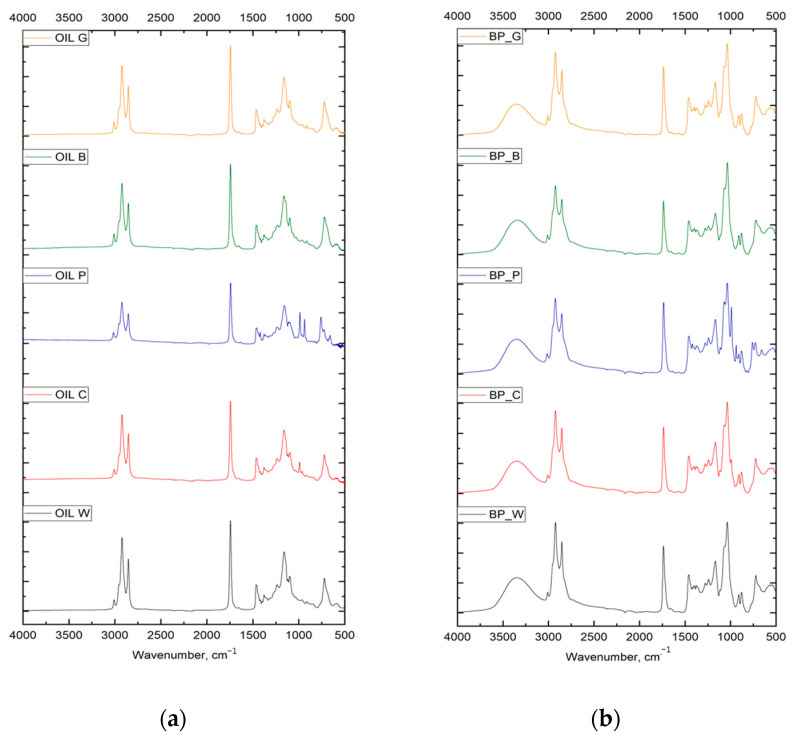
FTIR spectra of oils (**a**) and biopolyols (**b**).

The chemical structure of the oils and the biopolyols was analyzed by FTIR analysis. The spectra for the individual oils and biopolyols are shown in Figure 1a,b, respectively.

In the synthesis of polyurethane materials, the presence of hydroxyl groups is necessary. Hydroxyl groups are not present in the vegetable oils analyzed. The presence of such functional groups in biopolyols was confirmed by FTIR. The broad peak at 3340–3390 cm^−1^ corresponded to free hydroxyl groups in the biopolyols, and the absorption band at 1735–1745 cm^−1^ corresponded to C=O carbonyl bonds in ester groups.

The biopolyols were used to produce polyurethane foams. In the foams, 70% of petrochemical polyol was replaced with bio-based polyol. In the first stage of the research, the process of foaming polyurethane systems was analyzed. During the foaming process, a cellular structure is formed. It has a significant impact on the properties of PUR materials. During this process, the reactivity of PUR foam systems is determined by the dielectric polarization value. As the reaction progresses, the dielectric polarization decreases. This is due to the suppression of the mobility of hydroxyl groups and isocyanate groups, which are responsible for the formation of urethane groups and, consequently, polymer chains [29]. 

**Figure 2:**
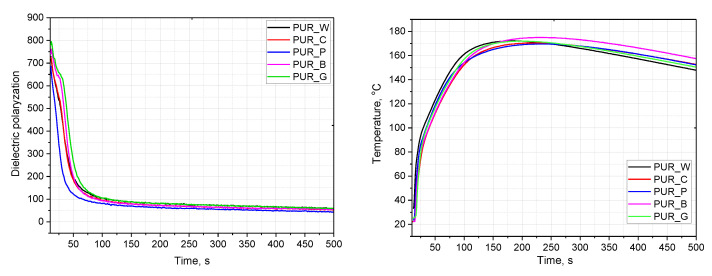
Changes in dielectric polarization and temperature during the foaming process of PUR systems.

The reactivity of PUR systems was changed by replacing petrochemical polyols with the biopolyols obtained from natural raw materials. The influence of the type of biopolyol on the changes in dielectric polarization during the foaming process of the PUR systems is shown in Figure 2.

Table 4 presents the characteristic foaming times of the PUR systems and the maximum temperatures during the foaming process.

The PUR systems modified with the biopolyols exhibited comparable reactivity levels. In the case of the PUR system containing the biopolyol from pomegranate seed oil, slightly greater reactivity was observed, as confirmed by a faster reduction in dielectric polarization. This is a very beneficial research result because the high reactivity of the biopolyols was confirmed, allowing the elimination of the catalyst from the PUR system. The high reactivity of the biopolyols was the result of the method of biopolyol synthesis. In the transesterification reaction of fruit seed oils, triethanolamine was applied. In addition to providing hydroxyl groups, it also has a lone pair of electrons in the nitrogen atom, which facilitates the catalysis of a PUR formation reaction. It is not possible to obtain a foam based only on petrochemical polyol RF551 without the use of a catalyst. A similar foam made only from petrochemical polyol was described in an earlier publication [29]. That foam material is made only from petrochemical polyol RF551, with the same amount of water and surfactant and using an analogous isocyanate as in these experiments. However, an amine catalyst was necessary to obtain it.

**Figure 3:**
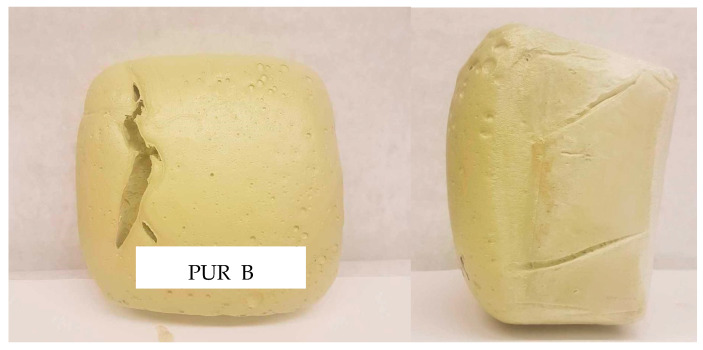
Structural defects in foam PUR_B.

The measured foaming times were comparable. Foam PUR_B had the highest value of the maximum temperature in the foam core during the foaming process. In the case of this material, structural defects in the form of cracks were also observed (Figure 3).

**Figure 4:**
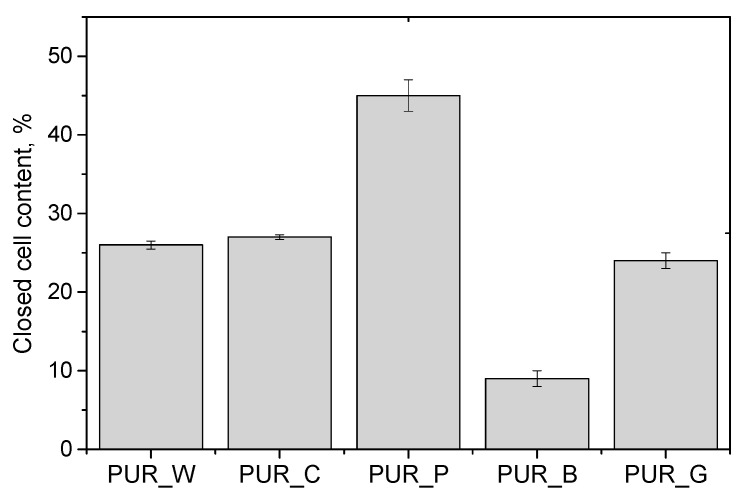
Closed cell content of PUR foams modified with biopolyols.

The cellular structure of foams has a significant impact on their physical–mechanical properties. If the foaming process occurs slower, the cells have a more uniform shape. Figure 4 shows the influence of the type of biopolyol on the closed cell content of the PUR foams.

A tendency to open cells was observed in the foams. The reference foam made only with petrochemical polyol had approximately 80 vol.% of closed cells [30]. It was observed that foam PUR_B was characterized by the lowest content of closed cells, which may be related to the highest foaming temperature causing an increase in pressure responsible for cell rupture [31]. 

The effect of cell opening may be related to the fact that the presence of an amine in the polyol structure accelerates the foaming reactions. The lack of organometallic catalysts that prefer catalyzing the gelation reactions results in an improper balance between gelation and foaming reactions. It was observed that the foams containing the biopolyol prepared from pomegranate seeds were characterized by the highest cell content. That was probably related to its highest viscosity, which affects the stabilization of cell formation during the foaming process. Nevertheless, the observed effect is extremely important because there is an increasing interest in foams with an open-cell structure. The use of a polyol component that affects the degree of cell opening allows the elimination of costly additives necessary when composing open-cell foam formulations.

**Figure 5:**
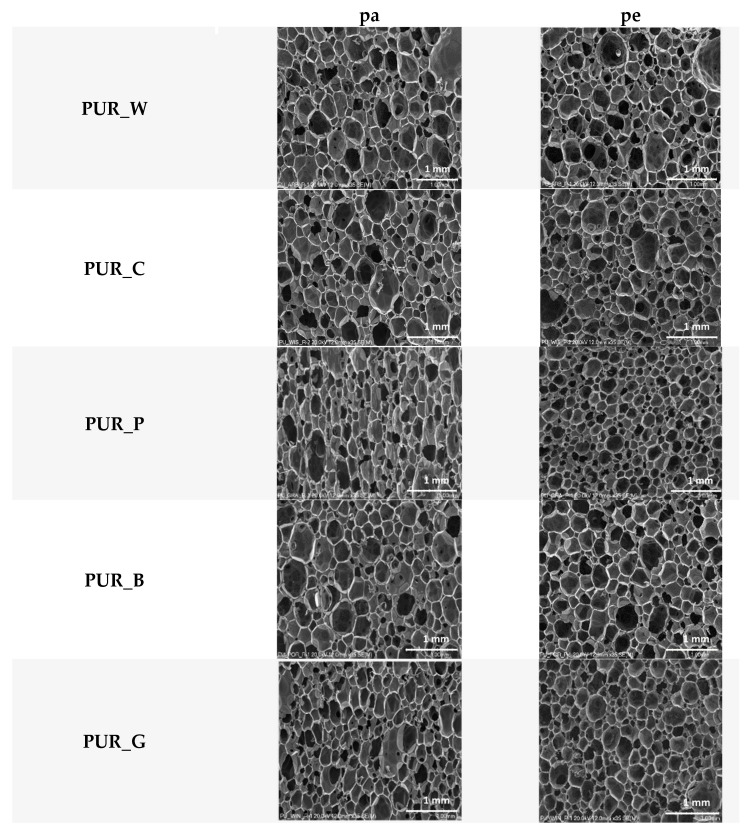
SEM micrographs of foam cross-sections parallel (pa) and perpendicular (pe) to the direction of foam growth.

SEM micrographs of the cross-section of the foams taken in directions parallel (pa) and perpendicular (pe) to the growth direction of the PUR system are shown in Figure 5.

It was found that foam PUR_P cells are characterized by the smallest sizes and the greatest elongation in the foam growth direction. Due to the rapid growth of the foam material, the cells elongate in the direction of growth [32]. The fine-cellular structure of the foam is desirable and has a beneficial effect on the mechanical properties of the biofoam. Foam PUR_B had the largest cells, which may be related to a slightly different foaming process compared to other foams. It is confirmed by the highest maximum temperature during foaming, the lowest content of closed cells and the lowest apparent density.

**Figure 6:**
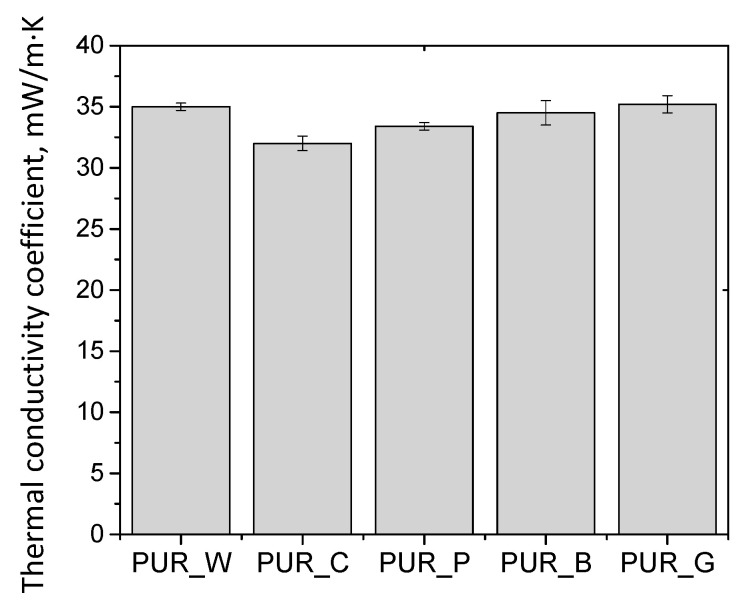
Thermal conductivity coefficients of PUR biofoams.

The thermal conductivity coefficient plays a key role in the case of insulating materials. This property is influenced by the content of closed cells as well as their shape. The values of the thermal conductivity coefficient and the closed cell contents of the foams obtained in our research are shown in Figure 4 and Figure 6, respectively.

The values of thermal conductivity coefficients of the foams were higher than those seen for typical thermal insulation closed-cell foams. However, this effect is related to a significant degree of cell opening. The reference foam made only from petrochemical polyol was characterized by a thermal conductivity coefficient of approximately 26 kW/m·K. In the case of open-cell PUR foams, the thermal conductivity coefficient is in the range of 38–42 mW/m·K [29,33]. Based on the test results, there was no significant relationship between the content of closed cells and the thermal conductivity coefficient for the PUR biofoams.

**Figure 7:**
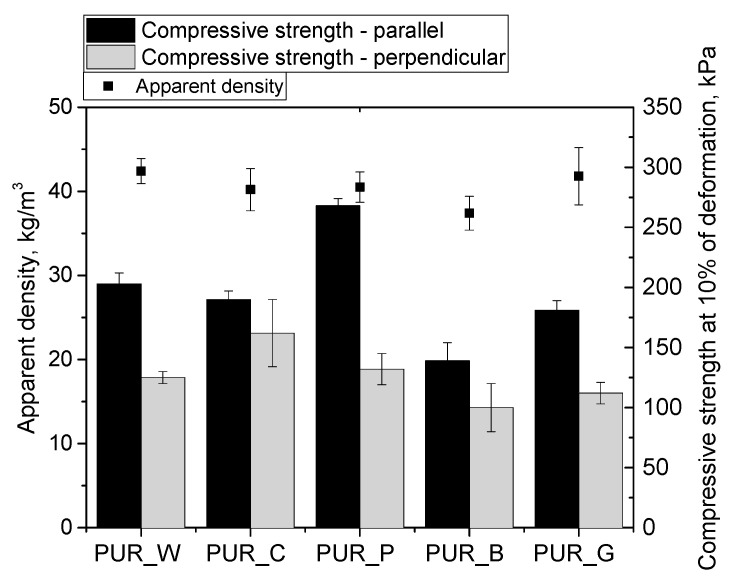
Apparent density and compressive strength of PUR biofoams derived from modified fruit seed oils.

The value of the apparent density of PUR foams has a significant impact on their mechanical properties. An increase in apparent density translates into an increase in compressive strength and stiffness of such foams. The main goal of the work was to obtain PUR materials with an apparent density of approximately 40 kg/m^3^. Figure 7 shows the apparent densities of the PUR foams and their mechanical strengths.

Among the PUR biofoams, foam PUR_P was characterized by the highest compressive strength in the parallel direction (268 kPa), which may be related to the highest content of closed cells and the smallest cell size. In the perpendicular direction, foam PUR_C is characterized by the highest durability, which is due to the shape of the cells, which are more isotropic compared to foam PUR_P where the cells were much more elongated in the direction of foam growth. In the case of foam PUR_B, the lowest values of compressive strength were obtained at 10% deformation, which is directly related to the lowest apparent density of this foam and the highest content of open cells. Despite the lowest mechanical strength, foam PUR_B was characterized by good dimensional stability. The reference foam was characterized by a higher apparent density of approximately 48 kg/m^3^, and as a result a higher compressive strength value, it reached up to 320 kPa [34].

Properties such as compressive strength and dimensional stability are closely related to each other. A change in temperature affects the gas pressure inside cells. It explains the difference between the atmospheric pressure outside a PUR foam and inside its cellular structure. The difference in these pressures must be less than the compressive strength of a PUR foam so that it can maintain its dimensional stability. Rigid PUR foams are dimensionally stable when their compressive strength is not lower than 100 kPa [34]. All the prepared biofoams had good dimensional stability.

Water absorption and brittleness were also tested. Foams as thermal insulation materials should have the lowest possible water absorption value. In Table 5 the influence of a given biopolyol on the water absorption and brittleness of the PUR foams is presented.

The use of biopolyols for the synthesis of PUR foams allowed materials with comparable brittleness and water absorption to be obtained. An exception is foam PUR_B whose brittleness exceeds 10% and water absorption approximately 2%. This effect is related to its lower apparent density and mechanical strength as well as the lowest content of closed cells compared to the other PUR materials.

## 4. Conclusions

Replacing 75% of petrochemical polyol with reactive biopolyols from fruit seed oil allowed the catalyst to be eliminated during the synthesis of rigid polyurethane foams. A tendency to open cells in the foams was observed, which resulted in thermal insulation materials with a thermal conductivity coefficient of approximately 32–33 mW/mK. This value is higher than for typical closed-cell foams and much lower than for foams with an open-cell structure. It was found that the maximum temperature during the foaming process of a polyurethane system containing biopolyol based on blackcurrant seed oil was the highest, which had a direct impact on the cellular structure of the resultant foam and related functional properties. In the case of the biofoam containing chemically modified pomegranate seed oil, a reduction in cell size and a higher content of closed cells were observed, which may be related to a higher viscosity of this biopolyol. It can be clearly stated that fruit seed oils, which have not been tested so far, after appropriate chemical modification, can constitute an alternative to oils such as rapeseed, soybean or palm oil commonly used until now in polyurethane foam synthesis. It has been observed that the addition of biopolyols obtained from plant oils acts as a surfactant. Therefore, it is important to select appropriate surfactants in the polyurethane system to obtain foams with a high content of closed cells.

## Figures and Tables

**Table 1:** Reagents used in the formulation of rigid PUR foams.

Biopolyol	Biofoam				
	PUR_W	PUR_C	PUR_P	PUR_B	PUR_G
	Mass of the Component, g				
BP_W	75	0	0	0	0
BP_C	0	75	0	0	0
BP_P	0	0	75	0	0
BP_B	0	0	0	75	0
BP_G	0	0	0	0	75
Surfactant	1.5	1.5	1.5	1.5	1.5
Water	3.0	3.0	3.0	3.0	3.0
Isocyanate	148.5	148.1	148.5	151.9	147.3

**Table 2:** Characteristics of oils derived from fruit seeds.

Oil	Source of Oil	I_val_, gI_2_/100 g	Mn, g/mol	Mw, g/mol	Ƞ, mPa·s
OIL_W	watermelon	118	865	871	53
OIL_C	cherry	137	859	865	55
OIL_P	pomegranate	101	881	886	200
OIL_B	blackcurrant	139	822	830	72
OIL_G	grape	133	859	865	46

**Table 3:** Characteristics of biopolyols.

Biopolyol	Source of Oil	OH_val_, mgKOH/g	Mn, g/mol	Mw, g/mol	Ƞ, mPa·s
BP_W	watermelon	361	279	471	165
BP_C	cherry	359	289	497	169
BP_P	pomegranate	361	335	558	413
BP_B	blackcurrant	381	276	473	160
BP_G	grape	355	281	484	157

**Table 4:** Characteristic foaming times of PUR systems and maximum temperatures during the foaming process.

	PUR_W	PUR_C	PUR_P	PUR_B	PUR_G
t_s_, s	<11	<11	<11	<11	<11
t_g_, s	21	22	20	20	25
t_d_, s	80	80	79	70	81
T_max_, °C	172	170	170	175	172
t _Tmax_, s	182	205	222	224	196

t_s_—start time, t_g_—gelation time, t_d_—dry time, T_max_—maximum temperature in the foam core, t_Tmax_—time to reach the maximum temperature.

**Table 5:** Water absorption and brittleness of PUR biofoams modified with fruit seed oils.

Foam	Brittleness, %	Water Absorption, %
PUR_W	5.3 ± 0.42	1.4 ± 0.31
PUR_C	7.2 ± 0.76	1.6 ± 0.35
PUR_P	5.5 ± 1.71	1.0 ± 0.15
PUR_B	10.2 ± 0.42	1.9 ± 0.27
PUR_G	4.1 ± 0.40	1.4 ± 0.35

## Data Availability

The data presented in this study are available on request from the corresponding author.
